# Quantifying Non-Gaussian Diffusion in Transient Microscopy Using Excess Kurtosis

**DOI:** 10.1021/acs.jpclett.5c03961

**Published:** 2026-02-12

**Authors:** Enrique Arévalo Rodríguez, Marc Meléndez, Jorge Cuadra, Ferry Prins

**Affiliations:** † Condensed Matter Physics Center, IFIMAC, Madrid 28049, Spain; ‡ Department of Condensed Matter Physics, 16722 Autonomous University of Madrid, Madrid 28049, Spain

## Abstract

Recent advances in transient microscopy have enabled high-resolution imaging of charge carrier dynamics. However, reliance on Gaussian fits to quantify population broadening can lead to misinterpretation when multiple species coexist. Transient scattering microscopy (TScM) provides a powerful alternative, yet its sensitivity to diverse species accentuates the limitations of traditional Gaussian fits. Here, we use TScM to visualize exciton transport in bulk transition metal dichalcogenides (TMDCs) and reveal that exciton populations exhibit non-Gaussian profiles by analyzing their excess kurtosis. Simulations incorporating anomalous diffusion reproduce these experimental observations and find that the signature of the kurtosis is distinct for coexisting populations and trap-dominated regimes. Additionally, we implement a discrete variable calculation to extract the variances which yields robust, consistent diffusivity values where Gaussian fits fail to do so. Our results establish kurtosis as a vital diagnostic parameter for identifying anomalous diffusion and demonstrate the necessity of moving beyond Gaussian approximations for analysis of TScM data.

## Graphical abstract

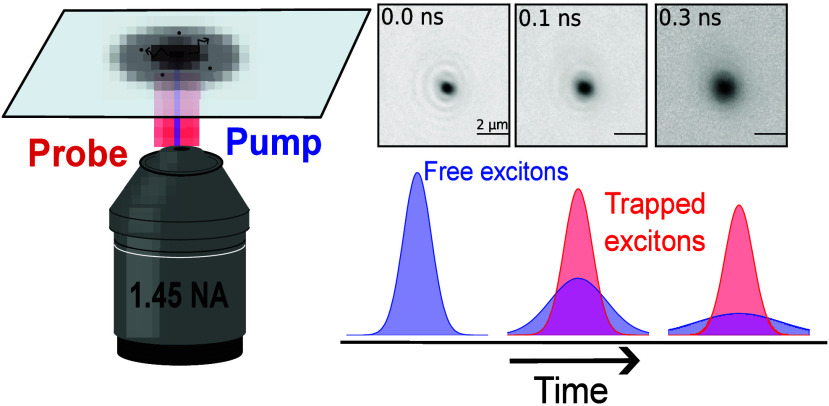

The study of carrier transport has advanced significantly in recent years with the emergence of a series of transient microscopy techniques, which enable imaging of energy carriers with nanometer-scale spatial and subnanosecond temporal resolution.^1–6^ These time-resolved measurements offer crucial insights into the various transport regimes that carriers may undergo during their lifetime, ranging from ballistic to diffusive and subdiffusive behaviors.^7–9^ By providing spatiotemporal maps of carrier dynamics, detailed information on how carrier populations evolve across complex energy landscapes can be obtained. Despite their relatively recent introduction, transient microscopy techniques have rapidly become an essential tool for the optoelectronic characterization of semiconductors.

Different transient microscopy techniques distinguish themselves mainly through their respective contrast mechanisms. Early examples include transient photoluminescence^10^ and transient absorption microscopy,^11^ both of which use diffraction limited excitation of a carrier population followed by spatiotemporal tracking of this population. In the case of transient photoluminescence microscopy (TPLM), radiative decay from the carriers is detected using spatially resolved time-correlated single photon counting or using streak camera approaches.^12^ Transient Absorption Microscopy (TAM) is a pump–probe technique that uses a spatially resolved probe beam to detect changes in the absorptivity of the material in the presence of carriers. Using absorption as a contrast mechanism has a major advantage in its ability to detect carriers independent of their radiative decay efficiencies.^13–15^ However, transient absorption microscopy has also typically inferior signal-to-noise ratios, requiring high excitation powers to obtain meaningful data.

More recently, transient microscopy based on interferometric scattering was reported.^16,17^ While related to Transient Absorption Microscopy, Transient Scattering Microscopy (TScM, also sometimes referred to as stroboSCAT) bases its contrast on small changes in the refractive index of a material in the presence of carriers, rather than changes in the absorptivity. Analogous to traditional interferometric scattering (iSCAT),^18^ TScM relies on the interference between scattered probe with the reflection at the glass-substrate interface to achieve higher signal-to-noise ratios as compared to absorption based techniques. Several studies on different material systems have since then been reported, including metal halide perovskites,^16^ super atomic semiconductors^7^ and few layer transition metal dichalcogenides (TMDCs).^19,20^

Importantly though, sensitivity to a large variety of populations can complicate the interpretation of the results. Extracting quantitative information from transient microscopy relies on the extraction of the change in variance of the excited state population, given by Δσ^2^ = σ­(*t*)^2^ – σ(0)^2^. To determine the variance, most Transient Microscopy studies fit the different time-slices to a Gaussian function.^21–24^ However, as a number studies have shown, complex dynamics can emerge when multiple mobile species are present,^9,25,26^ in the presence of higher-order recombination, or when subpopulations of trapped and free carriers dynamically interchange with each other. Importantly, in TScM, depending on the type of population and its corresponding effect in the refractive index of the material, both positive and negative contrast can be obtained, in some cases both effects can even coexist.^27,28^ Careful evaluation of the spatiotemporal evolution in the presence of the coexistence of multiple populations is therefore critical and requires more accurate quantitative analyses that go beyond Gaussian approximations of the carrier distribution.

Here, we employ Transient Scattering Microscopy (TScM) to investigate exciton transport in bulk tungsten diselenide (WSe_2_). Our data reveals significant deviations from pure Gaussian diffusion, which we quantify by means of the kurtosis of the exciton population. The temporal evolution of the excess kurtosis shows a transition from heavy tailed distributions at the beginning to short tailed at the end. Repetition rate dependent measurements show that the former is the result of a surviving population from previous laser pulses, while numerical simulations indicate that the late time dynamics is best described by excitons that encounter shallow traps as they diffuse through the material. Furthermore, by means of power-dependent measurements we demonstrate that Meitner–Auger recombination reduces excess kurtosis in the early times. Finally, as traditional Gaussian analyses fail to accurately describe the population evolution, we introduce an alternative and more reliable method to directly extract the spatial variance (and thus the diffusivity) from the distribution. Our work establishes kurtosis as a reliable metric to evaluate the signatures of different transport dynamics before making assumptions about the spatial distribution and its temporal evolution.

We perform transient scattering microscopy (TScM) measurements on multilayer tungsten diselenide (WSe_2_) flakes (>10 layers), mechanically exfoliated from commercially obtained large crystals of WSe_2_ and transferred to glass coverslips (see experimental methods section for details). TScM is performed using a near-diffraction-limited excitation laser (405 nm) as pump, whereas the probe (780 nm) is focused in the back focal plane of the objective to obtain wide field illumination. Images of the reflected probe are collected using a CMOS camera (FLIR BFS-U3–28S5M-C), a schematic of the optical setup in shown in Figure[Fig fig1]a. Synchronization between the CMOS and the laser driver with electronic delay (Picoquant Sepia PDL 828) allows us to record images of the excitation populations at different pump–probe delay times. Consecutive pump ON and pump OFF images are acquired and subsequently divided to generate differential images in the form *diff* = *ON*/*OFF* – 1. Figure 1b shows a series of resulting differential images for different pump–probe delays at an excitation fluence of 16 μJ/cm^2^ and 5 MHz laser repetition rate, with each image normalized to its maximum (negative) signal intensity. The negative intensity of the signal indicates a reduction in the reflectivity of the material upon photoexcitation, consistent with a reduced refractive index upon depletion of the ground-state carriers^2^.

**Figure 1:**
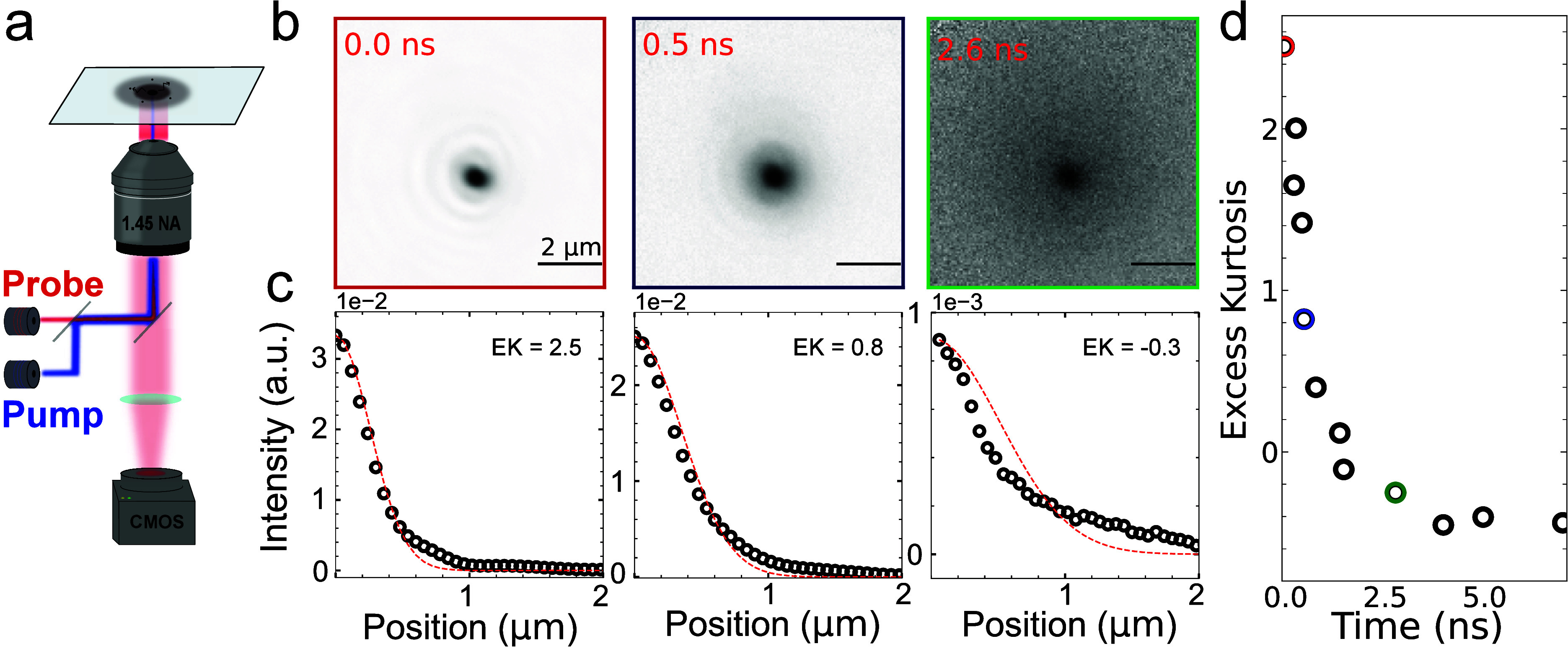
(a) Schematic of the transient scattering microscopy setup. (b) Transient scattering differential images taken at different pump–probe time delays. (c) Corresponding azimuthally averaged profiles of the pictures shown in panel b, dark points are the resulting averaged data points while dashed red lines show the best Gaussian fit for the provided data. (d) Excess kurtosis as a function of time for the complete data set shown in panel b, kurtosis is calculated using the full 2D image for each time delay to increase SNR.

The differential images reveal two contrast sources with distinct spatiotemporal dynamics: a fast-moving population that exhibits clear spatial broadening over time, and a slow-moving, long-lived population that remains largely stationary. As such, the time-dependent spatial distribution of the exciton population exhibits significant deviations from a two-dimensional Gaussian profile, deviating from purely Gaussian diffusion, complicating the quantitative analysis of the exciton diffusion dynamics. Figure 1c presents azimuthal averaged profiles of the exciton density at selected time delays shown in Figure 1b, along with their respective optimal Gaussian fits (dashed lines). At early times, the Gaussian fit fails to capture the heavy tails of the distribution. In contrast, as time goes by, the heavy tails of the distribution gradually vanish, at 0.3 ns the Gaussian fit does a good job of at fitting the profile, but at later times the tails become shorter than expected, leading to a short tailed or flat-topped distribution. We can describe the deviation from Gaussian shapes by calculating the kurtosis of the distribution as a function of time (see Methods for details). Within this definition, a perfect Gaussian distribution has an excess kurtosis of 0, while positive and negative values represent heavy tailed and flat-topped distributions, respectively. Results of the excess kurtosis for each differential image show a consistent temporal evolution, where the excess kurtosis starts positive and becomes smaller as time passes, stabilizing after reaching a negative value of around −0.5, as shown in Figure 1d.

To better understand the origin of the early excess kurtosis, we performed TScM at different laser repetition rates varying from 500 kHz to 10 MHz while maintaining the same fluence (65 μJ/cm^2^). Figure 2a shows the temporal evolution of the excess kurtosis for selected repetition rates. We observe that the early excess kurtosis drops significantly with decreasing repetition rate, going from values >2 for 10 MHz, indicating a heavy tailed distribution, to an excess kurtosis <1 for 500 kHz, indicating a much more Gaussian-like distribution. The decreasing excess kurtosis with decreasing repetition rate suggests that the source of the early positive kurtosis lies in a surviving population from previous laser pulses. This residual contrast appears as a secondary population that merges with the newly generated excitons by the subsequent pump pulse. This effect vanishes when the time between pulses is sufficiently long, allowing long-lived states to relax. This hypothesis is supported by calculations (SI section 1), which demonstrate that the sum of two Gaussian populations can produce a combined distribution with positive excess kurtosis.

**Figure 2:**
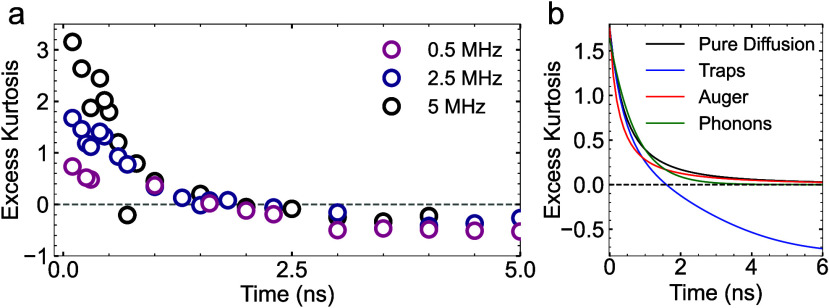
(a) Excess kurtosis as a function of time for selected data sets acquired with different laser repetition rates. (b) Numerical simulations for different dynamics representing the corresponding evolution of the excess kurtosis as a function of time. Black corresponds to pure Gaussian diffusion with no other effects, the red line represents the dynamics when Meitner–Auger recombination is present, the blue line shows the evolution of the kurtosis for dynamics dominated by trap states and the green line the calculation for a combination of noninteracting heat and exciton populations.

Having established the origin of the positive kurtosis at early times, we now turn to the spatiotemporal dynamics at longer times. All the results presented thus far exhibit a consistent trend where the excess kurtosis tends to negative values, stabilizing around −0.5. This behavior contrasts with the expectation for Gaussian diffusion. If we consider pure Brownian motion, any initial population, independent of the initial shape of the distribution, will tend to a value of 0 excess kurtosis with time. This behavior is confirmed by numerical simulations, as shown in Figure S3. Consequently, the time-dependent evolution toward a negative excess kurtosis at long times is a direct indication of more complex dynamics than simple Gaussian diffusion through Brownian motion.

Negative excess kurtosis is indicative of a flat-top distribution and has in the past been associated with Meitner–Auger recombination.^29,30^ As Meitner–Auger recombination occurs more efficiently at high exciton densities, the resulting faster decay at the center of the population indeed leads to a flattening of the distribution. Crucially though, the effect of Meitner–Auger recombination is limited to early times, diminishing quickly as the exciton density reduces as a result of both population decay and diffusion processes. As time goes by, Gaussian diffusion would again tend to a value of 0 excess kurtosis, as confirmed by numerical simulations (see red solid line in Figure 2b).

We now turn to the possibility of multiple mobile species causing negative excess kurtosis. As mentioned, TScM is sensitive not just to excitons or free carriers, but also to phonon populations. Phonons are generated as photoexcited carriers undergo bandedge relaxation in the first ps after excitation. The spatiotemporal evolution of the contrast is then a combination of both fast-moving short-lived excitons and slow-moving long-lived phonons. As time goes by, this would lead to a short-lived heavy tailed distribution caused by the fast outward moving excitons, leaving the slow moving phonons behind. With time, however, the long-lived phonon population with normal Gaussian diffusion would take over, once more leading to a negligible excess kurtosis. Simulations of this scenario are represented by the green solid line in Figure 2b). Indeed, at early times, a small prolongation of the positive excess kurtosis is observed, decaying to 0 as time goes by.

A distinct scenario with multiple mobile species is the presence of shallow traps. The presence of trap states has been shown to significantly affect the spatiotemporal dynamics of the population in transient microscopy measurements.^1,9,31^ In contrast to copropagating populations of excitons and phonons, shallow traps lead to a dynamic interchange between free and trapped excitons through consecutive trapping and detrapping events. Importantly, free excitons have a relative fast decay compared to trapped excitons. The less time excitons have resided in traps, the faster they move and the shorter their lifespans. This scenario indeed leads to a short-tailed (flat-topped) distribution with negative excess kurtosis, as demonstrated by our simulations (solid blue line in Figure 2b).

From our simulations, we can therefore conclude that the negative excess kurtosis is likely the result of the presence of shallow traps. The long-lived population responsible for early positive kurtosis could have the same origin, as traps cause longer-lived populations that can survive until the next laser pulse arrives.^26^ Importantly though, long-lived phonon populations provide an alternative explanation for the early positive kurtosis. Our analysis highlights the challenge that sensitivity to different carriers brings and the importance of kurtosis as an indicator of non-Gaussian diffusion dynamics.

While it is clear that long-lived states are the dominant factor in determining the evolution of excess kurtosis, Meitner–Auger recombination is known to play an important role in TMDCs at higher excitation densities.^25,30^ To elucidate how Meitner–Auger recombination additionally affects the excess kurtosis in WSe_2_, we perform TScM for increasing laser fluences, varying from 16 μJ/cm^2^ to 136 μJ/cm^2^. These experiments were performed at a repetition rate of 5 MHz. As can be seen in Figure 3a, the increasing excitation density has a distinct effect on the kurtosis, reducing the early time excess kurtosis as fluence increases. This trend is qualitatively consistent with our numerical simulations (Figure 2b), which also show a rapid decrease in early time kurtosis. To corroborate that this trend originates from Meitner–Auger recombination, we calculated the total contrast intensity by integrating the signal across all pump–probe delays for each fluence. The results (Figure 3b) reveal a clear transition from linear growth at low fluences (<10 μJ/cm^2^) to a sublinear regime at higher fluences, indicating a saturating behavior characteristic of Auger-mediated exciton decay.

**Figure 3:**
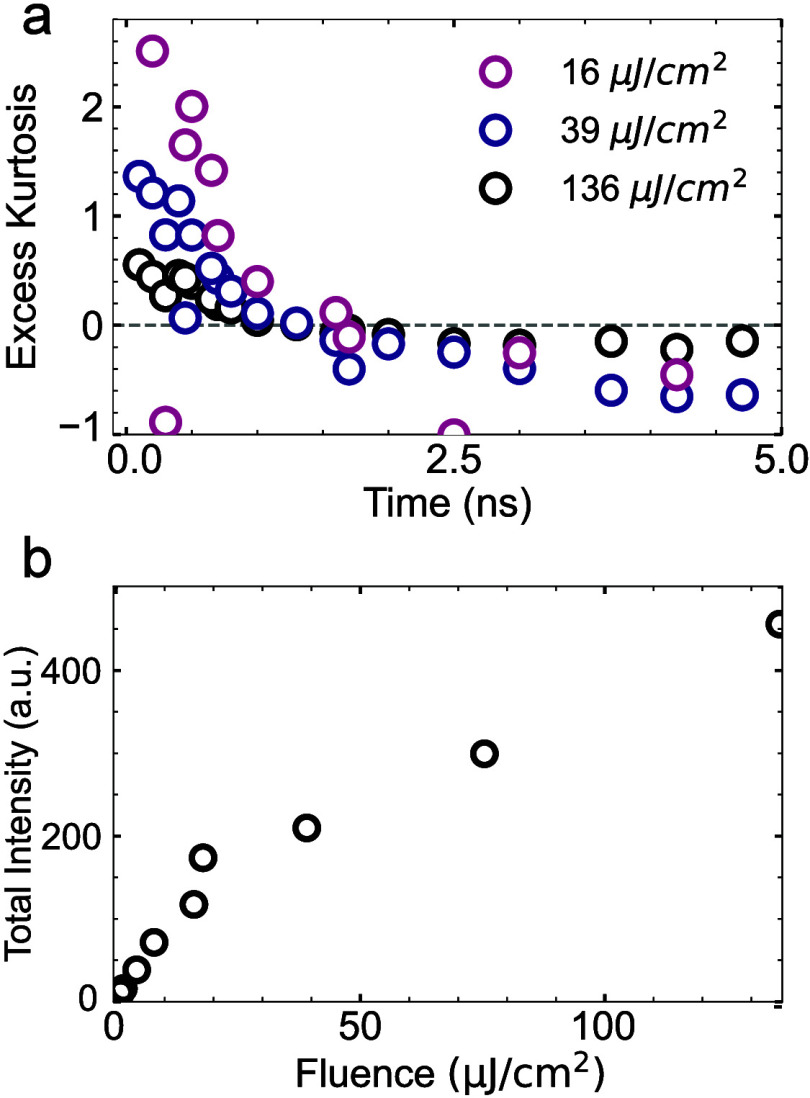
(a) Excess kurtosis as a function of time for different injected carrier densities. (b) Total calculated intensity contrast for the measured laser fluences.

A critical question is how strongly an analysis based on conventional Gaussian fits is affected by distributions with nonzero excess kurtosis. As it is common in the field,^14,32–34^ we perform Gaussian fits to one-dimensional line cuts from the differential images to calculate the change in variance as a function of time (Δσ^2^ = σ^2^(t) – σ^2^(t = 0)). As shown in Figure 4a, using Gaussian fits yields an unexpected dependence on the excitation power, with higher powers almost completely eliminating spatial broadening of the population. This result suggests clear limitations of Gaussian fitting when excess kurtosis is strongly time-dependent.

**Figure 4:**
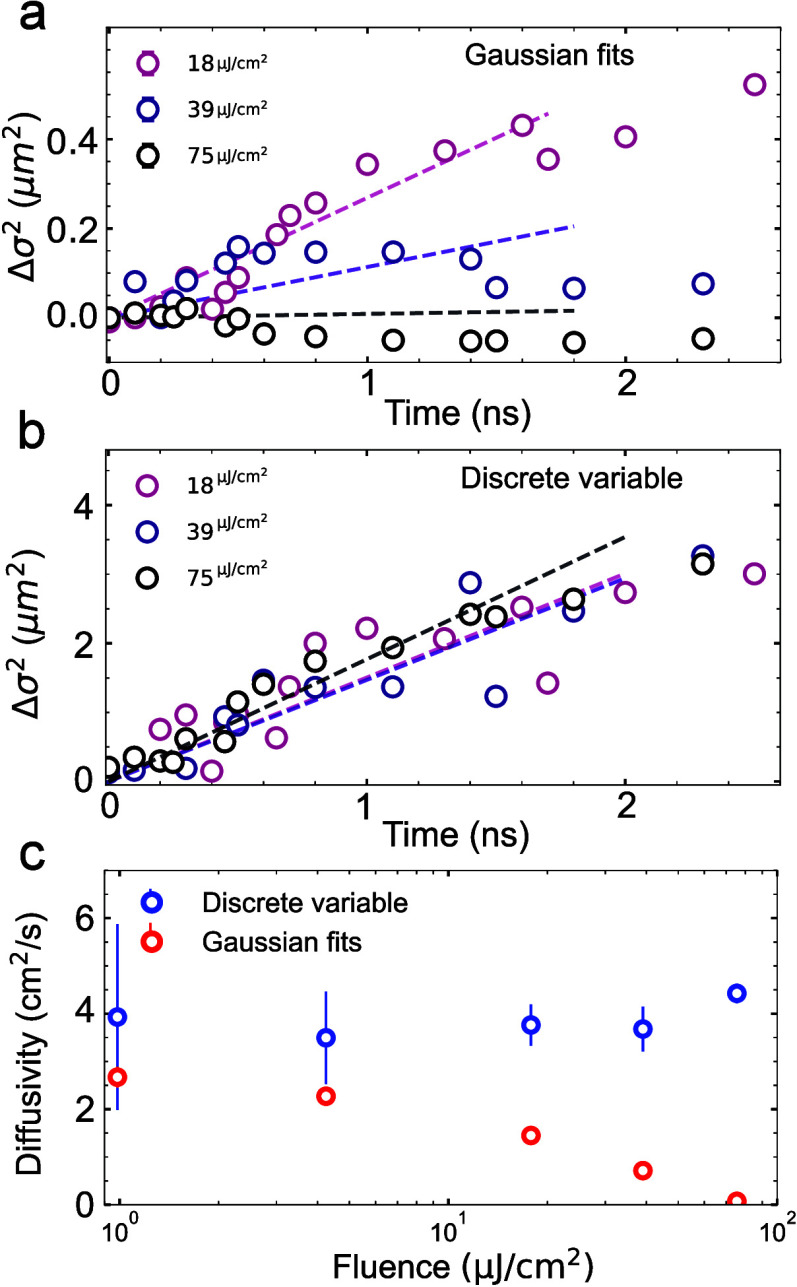
(a) Extracted values for Δσ^2^ using one-dimensional Gaussian fits from linecuts for selected injected energy densities. (b) Variances extracted from the same data sets as panel a using the discrete variable approach on the azimuthally averaged profiles. (c) Comparison of the calculated diffusivities for each method at different incident fluences, all diffusivities are obtained by performing a linear fit on the first 1.5 ns, before the sublinear diffusion regime.

To avoid making any assumptions on the shape of the carrier distribution, we can calculate the σ^2^ of the distribution by calculating the variance using a discrete variable approach:

$$\sigma^{2}=\frac{1}{I}\overset{n}{\underset{i}{\sum }}f_{i}{\left(x_{i}-\mu \right)}^{2}$$ 1

where *I* is the total intensity (sum of all the pixel intensities), *x*_*i*_ corresponds to the position of each pixel, *f*_*i*_ is the intensity value in that pixel and μ the expected value for x. In contrast to the Gaussian method, the discrete variable approach consistently recovers a comparable evolution for Δσ^2^ across all excitation powers (Figure 4b). Figure 4c summarizes the differences in the extracted diffusivity for both methods (see linear fits in Figure 4a and b and methods section for details). While Gaussian fits yield progressively smaller diffusivities at higher powers, the discrete variable method shows diffusivity values stable around 4 cm^2^/s. This value is comparable to previous reports on TMDCs.^35,36^ Our results validate the discrete variable approach as a more robust and consistent alternative to the more conventional Gaussian fitting.

We have presented the direct visualization of the exciton transport dynamics in bulk WSe_2_ using transient scattering microscopy. Our results show that the time-dependent exciton distributions deviate significantly from Gaussian diffusion and we quantify this deviation using kurtosis. Using repetition rate and fluence dependence of the excess kurtosis, we identify how trap states and Meitner–Auger recombination influence the observed population distributions at different points in time. We show how Gaussian fits can produce unphysical results for the diffusivity in the case of excess kurtosis and, alternatively, we propose the use of a discrete variable approach to avoid such artifacts. Our results establish the temporal evolution of the kurtosis as a sensitive diagnostic tool that can serve as an important first step in the analysis of Transient Microscopy data to identify non-Gaussian diffusion and avoid misrepresentation of the diffusivity of excited state carriers.

## Experimental Section

### Sample Preparation

Many-layer WSe_2_ flakes were mechanically exfoliated from a large crystal following reported techniques.^37^ Briefly, after exfoliation using adhesive tape, the flakes were deposited onto a transparent polydimethylsiloxane (PDMS) stamp. A three-axis manipulator was then used to transfer the WSe_2_ onto the desired glass substrate. The thickness of the flakes is estimated to be ∼ 20 layers by optical inspection.

### Transient Scattering Microscopy

For transient scattering measurements, laser diodes were used for the pump (405 *nm* Picoquant) and probe (780 *nm* Picoquant) sources, both lasers are controlled via the same driver (Picoquant Sepia II) which allows control of the delay between the two lasers via its electronic capabilities. After spatially filtering both lasers, the two are combined using a dichroic mirror and sent to the objective (Nikon Plan Apo 1.45 NA 100x). The reflected light is sent back to a CMOS camera after filtering out remaining pump light. The pump laser is modulated at 440 Hz, while the CMOS acquires pictures at twice the speed (880 Hz), thus acquiring consecutive images with and without the pump excitation.

We analyze our images by dividing subsequent ON/OFF image pairs, thus obtaining differential images *Diff* = *ON*/*OFF* – 1, which contain information about how much the reflection of the material changes between ON and OFF cases. A single experiment typically involves 4000 pairs for each pump–probe delay, the pairs are averaged together to reduce noise as much as possible. We note that these fluences are well below nonlinear optical effects for nanosecond pulsed excitation.^38,39^

### Derivation of the Kurtosis

Kurtosis is conventionally defined in one dimension as

$$k=E\left[\frac{{\left(x-\mu_{x}\right)}^{2}}{\sigma^{4}}\right]$$ 2

Although the kurtosis is well-defined in one dimension, for a bivariate distributions there are several ways to define the kurtosis. For isotropic distributions, where the variance in both directions is equal (σ_*x*_^2^ = σ_*y*_^2^), we can define the kurtosis in the form

$$k=E\left[\frac{{\left({\left(x-\mu_{x}\right)}^{2}+{\left(y-\mu_{y}\right)}^{2}\right)}^{2}}{\sigma^{4}}\right]$$ 3

where *E* is the distribution function and μ_*x*_ and μ_*y*_ are the expected values for the *x* and *y* positions, the value of σ^2^ is calculated using the discrete variable approach. It is worth noting that for this definition the kurtosis for a Gaussian distribution is eq 2 instead of the typical eq 3 for the one-dimensional case. With the exception of the data presented in Figure S9, where kurtosis is calculated one-dimensionally for azimuthally averaged profiles and *EK* = *k* – 3, all excess kurtosis values are calculated from the two-dimensional distributions as *EK* = *k* – 2.^40^

## Supplementary Material





## Data Availability

Codes to load and analyze the data and produce all figures contained in the manuscript are available at https://github.com/EaRodriguez3/TM_kurtosis. The data that support the findings of this study are openly accessible through the following link: https://doi.org/10.21950/2TSJNP.

## Notes

Additional numerical simulation details and results; calculation of the excess kurtosis for the sum of two Gaussian profiles; alternative double Gaussian fit analysis; additional experimental details including photographs of the studied flake in brightfield and iSCAT mode and the pump beam (PDF)
Transparent Peer Review report available (PDF)
