# Supplementary material for: Quantifying Non-Gaussian Diffusion in Transient Microscopy Using Excess Kurtosis
Source: J Phys Chem Lett. 2026 Feb 12;17(8):2479–84. doi: 10.1021/acs.jpclett.5c03961 (PMC12951552; doi:10.1021/acs.jpclett.5c03961)
Supplement: Supplementary file 1 [file jz5c03961_si_002.pdf]

Supplementary information for: Quantifying non-Gaussian diffusion in transient microscopy using excess kurtosis.

Enrique Arévalo Rodríguez,[†,†] Marc Meléndez,[‡,†] Jorge Cuadra,[‡,†] and Ferry Prins[*,‡,†]

†*Condensed Matter Physics Center, IFIMAC, Madrid, Spain*

‡*Department of Condensed Matter Physics, Autonomous University of Madrid, Madrid, Spain*

E-mail: ferry.prins@uam.es

Numerical simulations.

In the main text. we performed numerical simulations of exciton and heat diffusion. These simulations numerically integrate the following coupled differential equations. which determine how free, $c_0(r,t)$, and trapped, $c_i(r,t)$ excitons propagate:

$$\frac{\partial c_0(r,t)}{\partial t} = D_0 \nabla^2 c_0 - (\nu_0 + \sum_{i=i}^{N} \lambda_i) c_0 + \sum_{i=i}^{N} \mu_i c_i + \rho c_0^2 \tag{1}$$

$$\frac{\partial c_i(r,t)}{\partial t} = \lambda_i c_0 - (\mu_i + \nu_i) c_i \tag{2}$$

In these equations, D_0 is the exciton diffusivity, ν_0 the free exciton recombination rate, λ_i the trapping rate into trapped state i, μ_i the corresponding detrapping rate, ν_i the trapped

state recombination rate and ρ the Meitner-Auger recombination coefficient. The main text shows results for simulations in four different scenarios; pure diffusion, trap states, Auger and phonons, for each of these a different combination of the active terms is used.

For simulations (Figure 2b of the main text) incorporating only pure diffusion these are the only terms taken into account, and the positive excess kurtosis is fixed by changing the shape of the starting distribution to better match the experimental observation.

In the case of Auger recombination, we incorporate exclusively the non linear term (ρc_0^2). Similarly to before, the starting excess kurtosis is manually adjusted to fit the experiments.

For the simulations incorporating trap states, we introduce trapping ($-\lambda_1 c_0$) and de-trapping ($+\mu_1 c_1$) events, thus incorporating the second coupled differential equation (eq.2). Again, the excess kurtosis at the beginning is manually adjusted.

Lastly, for phonon transport, the model maintained the pure diffusion equation but we introduced a second non-interactive population with a lower diffusivity and longer lifetimes. these are chosen to reproduce experimental results. The total population calculated as the sum of both of these, independent, distributions. Crucially, in this case, instead of adjusting the positive excess kurtosis at early times we tune the exciton-phonon ratio to match it.

Importantly, all the simulations generate one-dimensional profiles as such, to calculate the variances and kurtosis from the simulated distributions we use equations 1 and 3 of the main text respectively.

Excess kurtosis for the sum of two Gaussian profiles.

In Figure 2 of the main text, we show that positive values of the early excess kurtosis may be caused by the overlap of two coexisting populations, a main population generated by the excitation beam and a surviving population originating from previous laser pulses. It is possible to mathematically prove that the combination of two populations will lead to positive values of the excess kurtosis.

Let us assume a total population described as $p(x) = ap_1(x) + bp_2(x)$, where p_1 and p_2 are the two coexisting populations, $a, b \geq 0$ and $a + b = 1$. To find the kurtosis, we first need to calculate the variance σ^2.

$$\sigma^2 = \int_{\infty}^{\infty} p(x)x^2dx = a\int_{\infty}^{\infty} p_1(x)x^2dx + b\int_{\infty}^{\infty} p_2(x)x^2dx = a\sigma_1^2 + b\sigma_2^2 \tag{3}$$

As the mean of $p(x)$ equals zero, the kurtosis can be written as:

$$\begin{aligned} K = \frac{E[x^4]}{E[x^2]^2} = \frac{E[x^4]}{(a\sigma_1^2 + b\sigma_2^2)^2} = \frac{\int_{\infty}^{\infty} p(x)x^4dx}{(a\sigma_1^2 + b\sigma_2^2)^2} \\ = \frac{a\int_{\infty}^{\infty} p_1(x)x^4dx + b\int_{\infty}^{\infty} p_2(x)x^4dx}{(a\sigma_1^2 + b\sigma_2^2)^2} = \frac{aK_1\sigma_1^4 + bK_2\sigma_2^4}{(a\sigma_1^2 + b\sigma_2^2)^2} \end{aligned} \tag{4}$$

where K_1, K_2 are the kurtosis for the two Gaussian functions, thus they equal 3. For $a = 0.95$ (newly generated population) and $b = 0.05$ (surviving population), the variances can be estimated to be $\sigma_1^2 = 2$ for the short-lived population and $\sigma_2^2 = 6$ for the long-lived population. In this case:

$$K = 3 * \frac{0.95 * 2^2 + 0.05 * 6^2}{(0.95 * 2 + 0.05 * 6)^2} = 3.47 \tag{5}$$

leading to a positive value of excess kurtosis.

In the main text we also introduced the possibility of phonons (heat) as the source of the large positive excess kurtosis as early times, however, numerical simulations showed that they could not be responsible for the features at long times. It is also possible to prove that the excess kurtosis at long times for two coexisting populations will not drop lower than 0 unless one of them is not Gaussian.

Starting from a two-dimensional analogue of Eq.4:

$$K = \frac{E[r^4]}{E[r^2]^2} = \frac{aK_1\sigma_1^4 + bK_2\sigma_2^4}{(a\sigma_1^2 + b\sigma_2^2)^2} \tag{6}$$

Where $r^2 = x^2 + y^2$- Assuming these two population propagate following Gaussian diffusion, that is, $\sigma_i = 4D_i t$ and $K_1 = K_2 = 2$

$$K = 2\frac{aD_1^2 + bD_2^2}{(aD_1 + bD_2)^2} \tag{7}$$

Where a and b are the fraction of the total population $N(t)$, thus $a = \frac{N_1(t)}{N(t)}$ and $b = \frac{N_2(t)}{N(t)}$. Substituting in eq7:

$$K = 2\frac{(N_1D_1)^2 + (N_2D_2)^2 + N_1N_2(D_1^2 + D_2^2)}{(N_1D_1)^2 + (N_2D_2)^2 + 2N_1N_2D_1D_2} \tag{8}$$

Since $D_1^2 + D_2^2 > 2D_1D_2$, the numerator is larger than the denominator and $K(t) > 2$ for all points in time.

Double Gaussian analysis

An alternative to the kurtosis analysis is an evaluation by separating the contrast arising from each population[1] and perform independent Gaussian fits to each population. However, reliably isolating two or more overlapping populations is often challenging. In our system at sufficiently high fluences, this separation becomes feasible via a simultaneous double Gaussian fit

The results of this analysis are presented in Fig. S1, alongside the discrete variable method. Figure S1a shows that the diffusivity extracted for the fast-moving population aligns closely with the value obtained from the discrete variable analysis. This agreement confirms that our discrete method is predominantly sensitive to the dynamics of the fast (exciton) population.

Furthermore, as seen in FigS1b, the double Gaussian fit captures the characteristic transition from a diffusive to a subdiffusive regime for the fast population. This implies that exciton transport is slowed down, an effect we attribute to interactions with trap states.

Consequently, the slower population (Fig. S1a) likely corresponds to heat diffusion. This assignment is supported by the fluence dependence: the contrast from the slow population increases with fluence, consistent with a larger thermal load. In contrast, trap-related signals would saturate at high fluences as states become filled.[2]

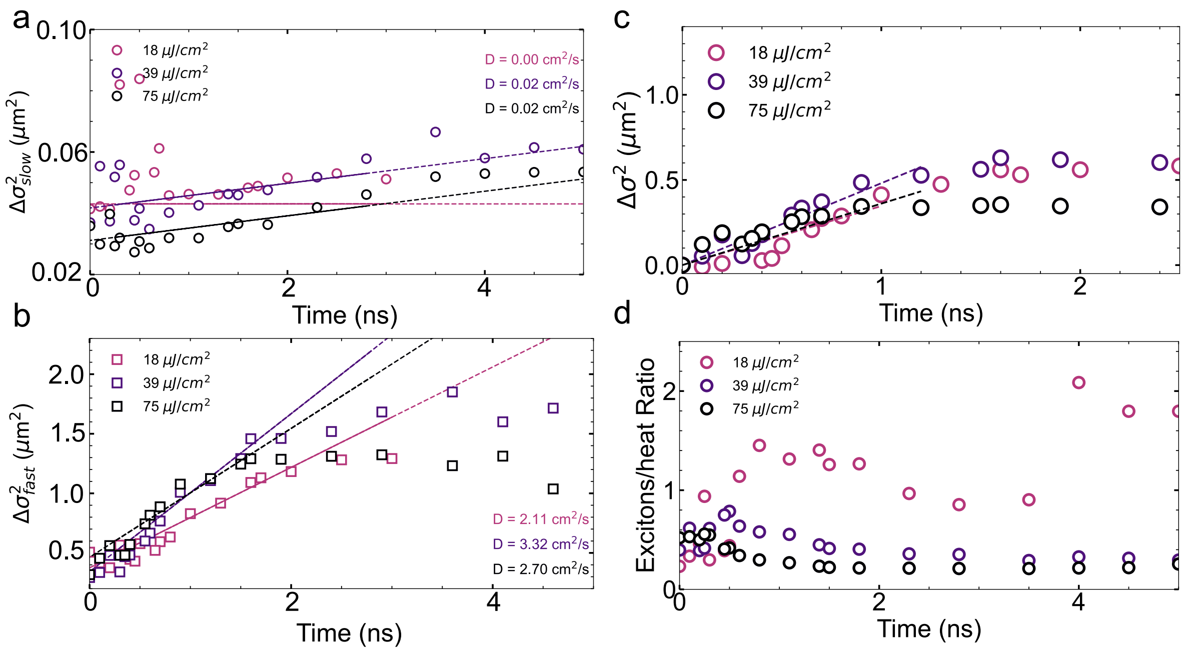

Figure S1: a. Slow component of the double Gaussian fit. b. Fast component of the Gaussian fit c. Variance extracted from the azimuthally averaged profiles using the discrete variable method d. Ratio of fast (excitons) vs slow (heat) components, the values are extracted from the double Gaussian fit

References

(1) Weaver, H. L.; Went, C. M.; Wong, J.; Jasrasaria, D.; Rabani, E.; Atwater, H. A.; Ginsberg, N. S. Detecting, Distinguishing, and Spatiotemporally Tracking Photogenerated Charge and Heat at the Nanoscale. *ACS Nano* **2023**, *17*, Publisher: American Chemical Society, 19011–19021.

(2) Seitz, M.; Magdaleno, A. J.; Alcázar-Cano, N.; Meléndez, M.; Lubbers, T. J.; Walraven, S. W.; Pakdel, S.; Prada, E.; Delgado-Buscalioni, R.; Prins, F. Exciton diffusion in two-

dimensional metal-halide perovskites. *Nature Communications* **2020**, *11*, Publisher: Nature Publishing Group, 2035.

Supplementary Figures

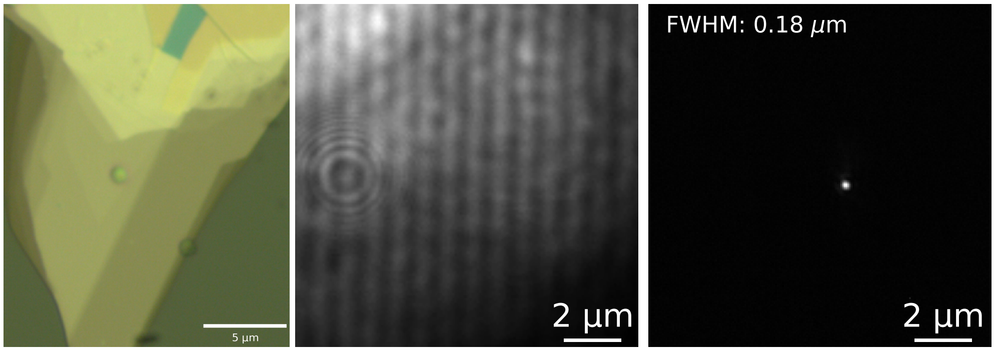

Figure S2: Brightfield image of the studied WSe_2 flake (left), iSCAT image (middle) and pump profile (right) with the calculated FWHM.

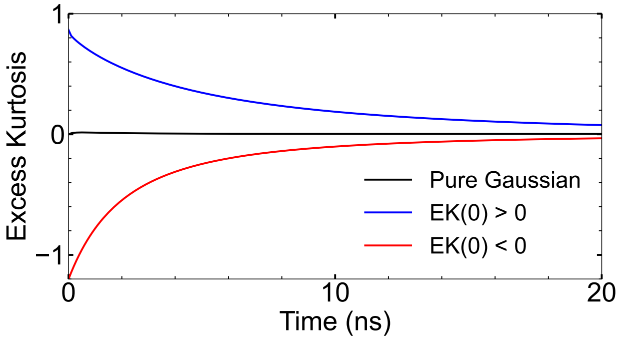

Figure S3: Evolution of the excess kurtosis for different values of initial excess kurtosis.

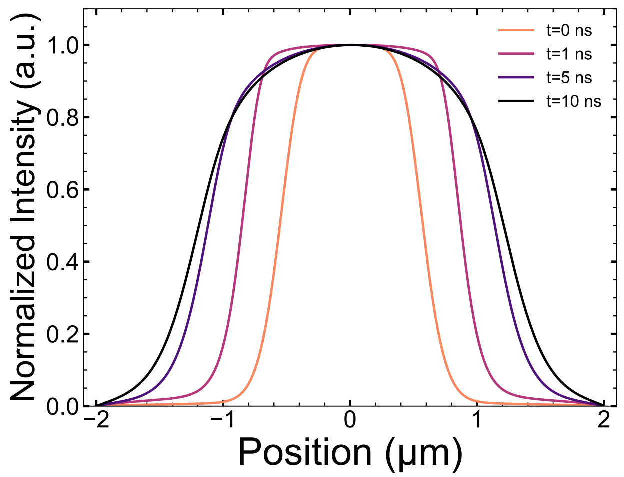

Figure S4: Numerical simulations for trap states showing the trend towards a thin-tailed distribution.

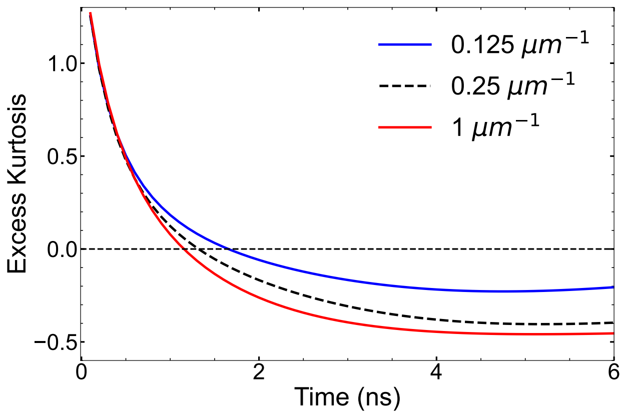

Figure S5: Numerical simulations for different values of the trap density.

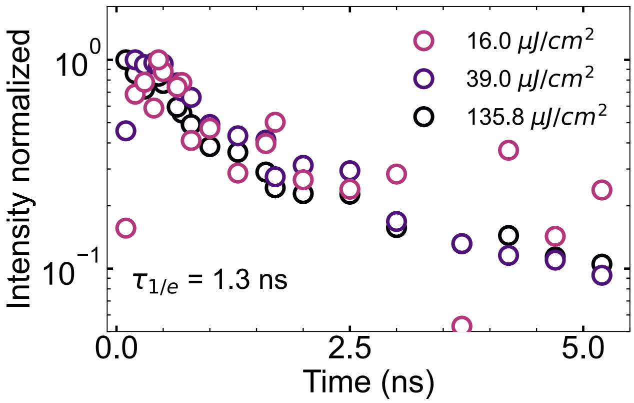

Figure S6: Lifetime traces of power-dependent data from Figure 3 in the main text.

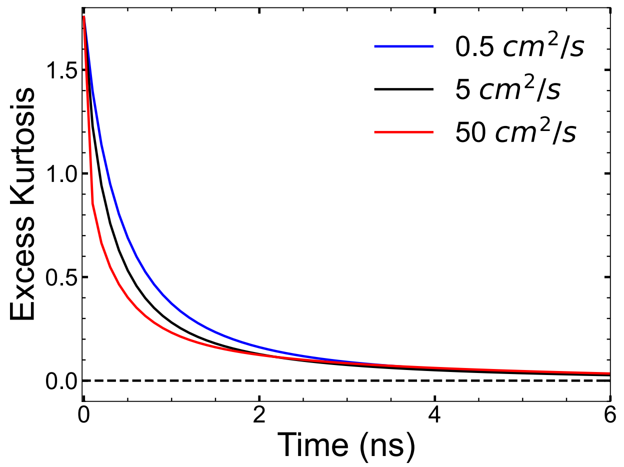

Figure S7: Evolution of the excess kurtosis for a distribution with increasing Meitner-Auger recombination rates.

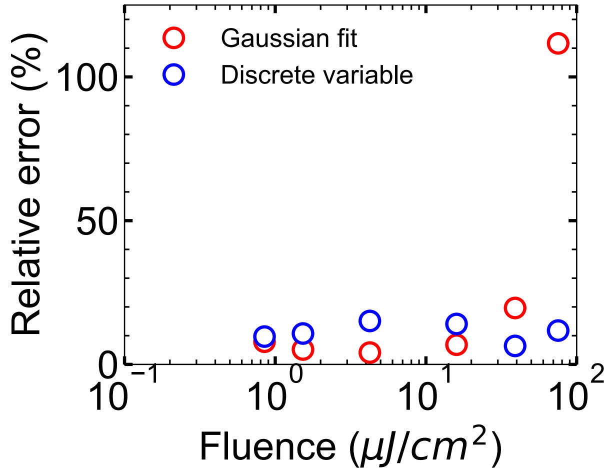

Figure S8: Relative error of the extracted diffusivities for the Gaussian fit and discrete variable methods. The error is calculated from the fit to a linear function as depicted in the main text.

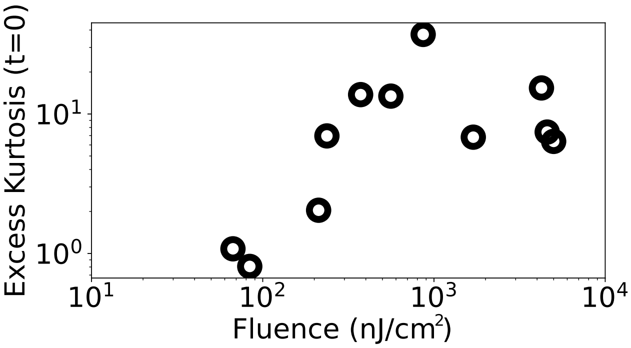

Figure S9: Excess kurtosis as a function of laser fluence. EK is calculated from the azimuthally averaged profiles to improve SNR in the low fluence measurements.
