# Supplementary material for: Quantifying Non-Gaussian Diffusion in Transient Microscopy Using Excess Kurtosis
Source: J Phys Chem Lett. 2026 Feb 12;17(8):2479–84. doi: 10.1021/acs.jpclett.5c03961 (PMC12951552; doi:10.1021/acs.jpclett.5c03961)
Supplement: Supplementary file 2 [file jz5c03961_si_003.pdf]

jz-2025-03961a.R1

Name: Peer Review Information for "Quantifying non-Gaussian diffusion in transient microscopy using excess kurtosis."

First Round of Reviewer Comments

Reviewer: 1

Comments to the Author

This manuscript presents a compelling and well-executed study on quantifying non-Gaussian diffusion in transient scattering microscopy (TScM) using excess kurtosis. The work is technically sound, methodologically rigorous, and makes a significant contribution to the field of carrier dynamics characterization.

The introduction of excess kurtosis as a quantitative diagnostic parameter for TScM data is a novel and powerful approach. Moving beyond the conventional, yet often limiting, Gaussian fitting paradigm addresses a critical gap in the analysis of complex, multi-species dynamics. The proposed discrete variable method for calculating variance directly from the distribution, without assuming its shape, is a robust and elegant solution that effectively overcomes the artifacts introduced by traditional fitting when kurtosis is non-zero. This methodological advancement is not merely incremental; it provides the community with a new, more reliable toolkit for extracting diffusivities from transient microscopy images. The authors successfully leverage kurtosis to deconvolve the spatiotemporal evolution of exciton populations in bulk WSe_2, revealing distinct dynamical regimes. The clear identification of early-time positive kurtosis arising from residual populations and late-time negative kurtosis indicative of trap-dominated dynamics provides deep physical insight. The fluence-dependent studies further disentangle the influence of Meitner-Auger recombination. These results convincingly demonstrate that kurtosis is a sensitive probe for identifying anomalous diffusion and the coexistence of different carrier species or processes, which are easily missed or misinterpreted with standard analysis. This work establishes a critical interpretive framework that will enhance

the accuracy and physical relevance of future transient microscopy studies across various material systems.

In summary, this manuscript introduces a timely and innovative analytical framework that advances the interpretation of transient microscopy data. The findings are important, clearly presented, and well-supported by both experiment and simulation. I have no major revisions to suggest and recommend acceptance for publication in its current form.

Reviewer: 2

Comments to the Author

This manuscript investigates non-Gaussian diffusion in transient absorption microscopy (TAM) via excess kurtosis analysis. The findings offer valuable insights into exciton diffusion dynamics and hold particular significance for future applications of this technique. However, several key issues should be addressed prior to publication:

1) The Gaussian fits presented in Figure 1c exceed the plot boundaries and overlap significantly, complicating visual assessment. More importantly, the fit at 2.6 ns appears to deviate notably from the experimental data, whereas the authors state that larger excess kurtosis—indicative of stronger non-Gaussian behavior—occurs at earlier times (as suggested in Figure 2). This apparent discrepancy between the fitted curves and the claimed temporal evolution of kurtosis should be clarified.

2) As a third-order nonlinear spectroscopic technique, TAM signals arise from the spatial overlap of the pump and probe beams. Given that both beams are typically Gaussian, the observed signal profile is expected to reflect the product of their intensity distributions. The manuscript should explicitly state whether this effect has been accounted for in the simulations and data fitting. Additionally, characterization of the experimental beam profiles should be provided, as this constitutes a fundamental assumption in the spatial analysis.

3) The authors attribute the observed excess kurtosis to residual excitons from preceding laser pulses. To evaluate this hypothesis, it would be helpful to know the exciton lifetime in the sample. Were measurements performed at a reduced repetition rate to minimize excitonic accumulation from the previous laser pulses? Demonstrating that the effect

vanishes (or diminishes) under conditions designed to avoid residual excitons would strengthen the interpretation.

4) At the fluence levels employed, non-resonant signals (e.g., from two-photon absorption or Kerr lensing) may contribute near time zero. Such contributions could distort the spatial profile of the transient absorption signal at early delay times. The authors should discuss whether these effects have been considered and how they might influence the reported kurtosis analysis.

5) Since signals are collected in a scattering manner, interference effects could potentially alter the apparent spatial profile, particularly at early times. A brief discussion of whether scattering-induced interference has been accounted for—or is expected to be negligible—would help readers assess the robustness of the spatial diffusion analysis.

Reviewer: 3

Comments to the Author

The authors propose a comprehensive data analysis method based on excess kurtosis to extract anomalous diffusion information from the non-Gaussian profiles in transient microscopy. The underlying physics is sound, and the data presented are sufficient to support the conclusions. However, significant formatting issues in the figures and manuscript must be addressed. Prior to publication in JPCL, all figures require careful revision, and the reference list must be corrected to meet the journal's style guidelines.

Author's Response to Peer Review Comments:

We thank the editor and reviewers for their time and comments on our manuscript. We have considered all the comments and addressed the concerns the reviewers mentioned. Please find our responses to each comment below.

Reviewer(s)' Comments to Author:

Reviewer: 1

Recommendation: This paper is publishable subject to minor revisions noted. Further review is not needed.

Comments:
This manuscript presents a compelling and well-executed study on quantifying non-Gaussian diffusion in transient scattering microscopy (TScM) using excess kurtosis. The work is technically sound,

methodologically rigorous, and makes a significant contribution to the field of carrier dynamics characterization.

The introduction of excess kurtosis as a quantitative diagnostic parameter for TScM data is a novel and powerful approach. Moving beyond the conventional, yet often limiting, Gaussian fitting paradigm addresses a critical gap in the analysis of complex, multi-species dynamics. The proposed discrete variable method for calculating variance directly from the distribution, without assuming its shape, is a robust and elegant solution that effectively overcomes the artifacts introduced by traditional fitting when kurtosis is non-zero. This methodological advancement is not merely incremental; it provides the community with a new, more reliable toolkit for extracting diffusivities from transient microscopy images. The authors successfully leverage kurtosis to deconvolve the spatiotemporal evolution of exciton populations in bulk WSe_2, revealing distinct dynamical regimes. The clear identification of early-time positive kurtosis arising from residual populations and late-time negative kurtosis indicative of trap-dominated dynamics provides deep physical insight. The fluence-dependent studies further disentangle the influence of Meitner-Auger recombination. These results convincingly demonstrate that kurtosis is a sensitive probe for identifying anomalous diffusion and the coexistence of different carrier species or processes, which are easily missed or misinterpreted with standard analysis. This work establishes a critical interpretive framework that will enhance the accuracy and physical relevance of future transient microscopy studies across various material systems.

In summary, this manuscript introduces a timely and innovative analytical framework that advances the interpretation of transient microscopy data. The findings are important, clearly presented, and well-supported by both experiment and simulation. I have no major revisions to suggest and recommend acceptance for publication in its current form.

We sincerely thank Reviewer 1 for their positive assessment of our manuscript and their effort and time reviewing our work. We are pleased they found it insightful and well written.

Reviewer 2.

This manuscript investigates non-Gaussian diffusion in transient absorption microscopy (TAM) via excess kurtosis analysis. The findings offer valuable insights into exciton diffusion dynamics and hold particular significance for future applications of this technique. However, several key issues should be addressed prior to publication:

We thank Reviewer 2 for their insightful comments. We would like to stress that our work is not based on Transient Absorption Microscopy, but rather on Transient Scattering Microscopy. While this may be a subtle difference in some respects, this distinction has important implications regarding some the of comments raised by the reviewer. To emphasize the difference between TAM and TScM, we have modified the following sentence in the introduction and included an explicit reference to the review paper of Ginsberg and Tisdale in which the differences are discussed in detail. The new sentence in the third paragraph now reads:

While related to Transient Absorption Microscopy, Transient Scattering Microscopy (TScM, also sometimes referred to as stroboSCAT) bases its contrast on small changes in the refractive index of a material in the presence of carriers, rather than changes in the absorptivity.

In the following we will further clarify the remaining points of the reviewer:

1. 'The Gaussian fits presented in Figure 1c exceed the plot boundaries and overlap significantly, complicating visual assessment. More importantly, the fit at 2.6 ns appears to deviate notably from the experimental data, whereas the authors state that larger excess kurtosis—indicative of stronger non-Gaussian behavior—occurs at earlier times (as suggested in Figure 2). This apparent discrepancy between the fitted curves and the claimed temporal evolution of kurtosis should be clarified.'

Indeed, the profile at 2.6 ns deviates from a Gaussian profile, exhibiting significant ***negative*** excess kurtosis. As discussed in the main text, while at early times a positive excess kurtosis is observed, this shifts to a negative excess kurtosis at later times. We have added the value of the EK to each panel in Figure 1c to clarify that the fit at 2.6 ns is expected to substantially deviate from the experimental data as it shows considerable negative excess kurtosis. The new figure now shows:

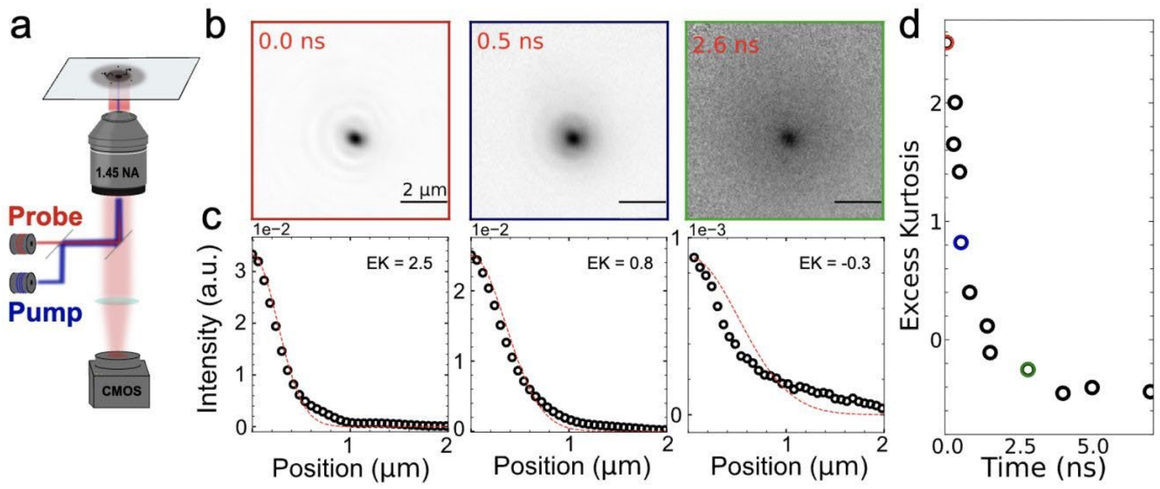

2. 'TAM signals arise from the spatial overlap of the pump and probe beams. Given that both beams are typically Gaussian, the observed signal profile is expected to reflect the product of their intensity distributions. The manuscript should explicitly state whether this effect has been accounted for in the simulations and data fitting. Additionally, characterization of the experimental beam profiles should be provided, as this constitutes a fundamental assumption in the spatial analysis'

We stress once more that TAM differs significantly from our implementation of transient scattering microscopy. While TAM indeed requires overlapping gaussian pump-probe profiles, TScM is able to operate using a wide-field probe configuration and only the pump is focused down with a Gaussian profile. Nevertheless, to further clarify the configuration of the pump and probe beams we have added the probe and pump profiles to figure S2 in the supporting information. The new figure now shows:

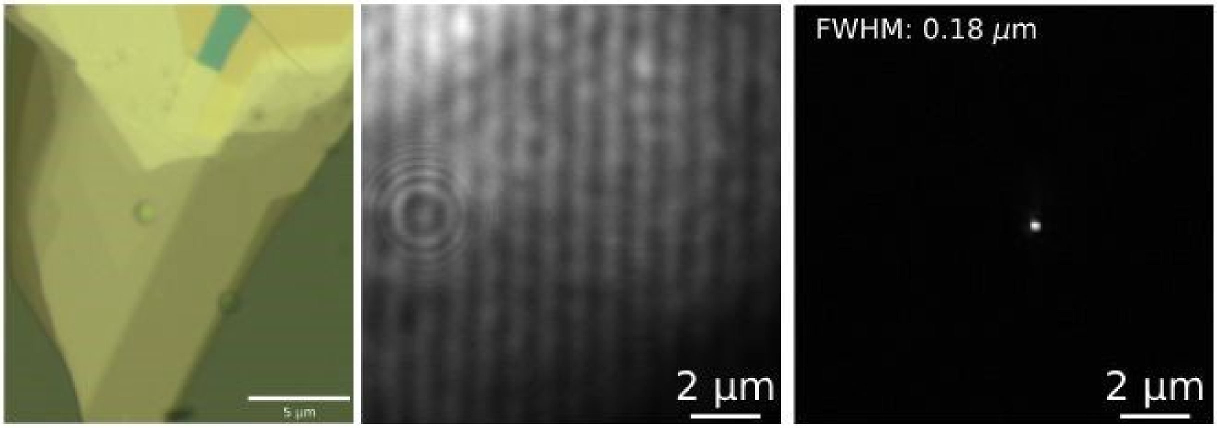

3.'The authors attribute the observed excess kurtosis to residual excitons from preceding laser pulses. To evaluate this hypothesis, it would be helpful to know the exciton lifetime in the sample. Were measurements

performed at a reduced repetition rate to minimize excitonic accumulation from the previous laser pulses? Demonstrating that the effect vanishes (or diminishes) under conditions designed to avoid residual excitons would strengthen the interpretation.'

We agree with Reviewer 2 that the value of the lifetime can provide valuable insight into the underlying physics, therefore, we have added the extracted lifetime of the decay traces in figure S6. Indeed, as they point out, repetition rate dependent measurements in Figure 2 of the main text show that the effect of positive excess kurtosis diminishes with reduced repetition rate.

4. 'At the fluence levels employed, non-resonant signals (e.g., from two-photon absorption or Kerr lensing) may contribute near time zero. Such contributions could distort the spatial profile of the transient absorption signal at early delay times. The authors should discuss whether these effects have been considered and how they might influence the reported kurtosis analysis.'

We thank the reviewer for this question regarding potential non-resonant contributions at early delay times. At our experimental fluence levels (~100 μJ/cm^2), non-resonant nonlinear optical effects such as two-photon absorption and Kerr lensing are expected to be negligible. Studies of nonlinear optical responses in transition metal dichalcogenides with nanosecond pulses have established that significant TPA and other third-order nonlinear effects require substantially higher fluences than employed in our experiments. Our fluence is approximately three to four orders of magnitude below the reported thresholds for observing TPA or Kerr nonlinearities in TMDC materials with nanosecond pulses. At these sub-threshold fluences, the transient absorption signal should be dominated by resonant excitonic processes rather than non-resonant nonlinear optical effects. We have added the following sentence and two references to the methods section of the manuscript:

We note that these fluences are well below non-linear optical effects for nanosecond pulsed excitation.[38,39]

38. Wang, G. et al. Understanding the linear and nonlinear optical responses of few-layer exfoliated MoS_2 and WS_2 nanoflakes: experimental and simulation studies. *Nanotechnology* **31**, 205702 (2020).
39. Dong, N. et al. Optical Limiting and Theoretical Modelling of Layered Transition Metal Dichalcogenide Nanosheets. *Sci. Rep.* **5**, 14646 (2015).

5. 'Since signals are collected in a scattering manner, interference effects could potentially alter the apparent spatial profile, particularly at early times. A brief discussion of whether scattering-induced interference has been accounted for—or is expected to be negligible—would help readers assess the robustness of the spatial diffusion analysis.'

Indeed, as explained in paragraph 3, the high CNR in TScM is obtained through interferometric scattering (analogous to iSCAT). To further clarify this, we have adapted the following sentence in paragraph 3:

'Analogous to traditional interferometric scattering (iSCAT), TScM relies on the interference between scattered probe with the reflection at the glass-substrate interface to achieve higher signal-to-noise ratios as compared to absorption-based techniques.'

Reviewer 3.

The authors propose a comprehensive data analysis method based on excess kurtosis to extract anomalous diffusion information from the non-Gaussian profiles in transient microscopy. The underlying physics is sound, and the data presented are sufficient to support the conclusions. However, significant formatting issues in the figures and manuscript must be addressed. Prior to publication in JPCL, all figures require careful revision, and the reference list must be corrected to meet the journal's style guidelines.

We thank Reviewer 3 for their comments. Following JPCL guidelines we have adjusted our figures to match the journal style better.

Once again, we would like to thank the reviewers and editor for their time. We believe the revised manuscript has benefitted a lot from the reviewers' suggestion to improve its clarity and impact. We hope the updated manuscript is suitable for publication in JPCL and that it meets your approval.

jz-2025-03961a.R2

Name: Peer Review Information for "Quantifying non-Gaussian diffusion in transient microscopy using excess kurtosis."

Second Round of Reviewer Comments

Reviewer: 2

Comments to the Author

The current version of manuscript has addressed all the reviewers' concerns and can be published as it is.

Author's Response to Peer Review Comments:

Dear Editor,

Thank you for your positive assessment of our manuscript, 'Quantifying non-Gaussian diffusion in transientmicroscopy using excess kurtosis' , for publication in Journal of Physcal Chemistry Letters.

We have carefully reviewed the editorial requests to facilitate a manuscript version up to the journal standards. The reviewed manuscipt has already been submitted to the submission system.

Once again, we would like to thank both you and the reviewers for the constructive feedback throughout the peer-review process, which has significantly improved our paper.

We look forward to seeing the paper published.

Best regards,

Enrique Arévalo Rodríguez
